# Numerical Simulations of Realistic Lead Trajectories and an Experimental Verification Support the Efficacy of Parallel Radiofrequency Transmission to Reduce Heating of Deep Brain Stimulation Implants during MRI

**DOI:** 10.1038/s41598-018-38099-w

**Published:** 2019-02-14

**Authors:** C. E. McElcheran, L. Golestanirad, M. I. Iacono, P.-S. Wei, B. Yang, K. J. T. Anderson, G. Bonmassar, S. J. Graham

**Affiliations:** 1 Physical Sciences Platform, Sunnybrook Health Sciences Institute, Toronto, Ontario M4N 3M5 Canada; 2 Department of Medical Biophysics, University of Toronto, Toronto, Ontario M5S 1A8 Canada; 3 Department of Biomedical Engineering, Northwestern University, Evanston, IL 60208 USA; 4 Division of Biomedical Physic, Office of Science and Engineering Laboratories, Center for Devices and Radiological Health, U.S Food and Drug Administration, Silver Spring, MD 20993 USA; 5 Athinoula A. Martinos Center For Biomedical Imaging, Department of Radiology, Massachusetts General Hospital, Charlestown, MA 02129 USA; 6 Harvard Medical School, Boston, MA 02115 USA

## Abstract

Patients with deep brain stimulation (DBS) implants may be subject to heating during MRI due to interaction with excitatory radiofrequency (RF) fields. Parallel RF transmit (pTx) has been proposed to minimize such RF-induced heating in preliminary proof-of-concept studies. The present work evaluates the efficacy of pTx technique on realistic lead trajectories obtained from nine DBS patients. Electromagnetic simulations were performed using 4- and 8-element pTx coils compared with a standard birdcage coil excitation using patient models and lead trajectories obtained by segmentation of computed tomography data. Numerical optimization was performed to minimize local specific absorption rate (SAR) surrounding the implant tip while maintaining spatial homogeneity of the transmitted RF magnetic field (B_1_^+^), by varying the input amplitude and phase for each coil element. Local SAR was significantly reduced at the lead tip with both 4-element and 8-element pTx (median decrease of 94% and 97%, respectively), whereas the median coefficient of spatial variation of B_1_^+^ inhomogeneity was moderately increased (30% for 4-element pTx and 20% for 8-element pTx) compared to that of the birdcage coil (17%). Furthermore, the efficacy of optimized 4-element pTx was verified experimentally by imaging a head phantom that included a wire implanted to approximate the worst-case lead trajectory for localized heating, based on the simulations. Negligible temperature elevation was observed at the lead tip, with reasonable image uniformity in the surrounding region. From this experiment and the simulations based on nine DBS patient models, optimized pTx provides a robust approach to minimizing local SAR with respect to lead trajectory.

## Introduction

The symptoms of several major neurological and psychiatric disorders such as Parkinson’s Disease^1^, essential tremor^2,3^, and dystonia^4,5^ are effectively relieved by deep brain stimulation (DBS). DBS devices consist of a pulse generator implanted in the chest or abdomen to deliver electrical pulses to specific deep brain nuclei through long, conductive leads, which are inserted during a minimally-invasive neurosurgical procedure. Precise localization of DBS electrodes is vital for effective treatment. Deviation of electrode position from the target reduces therapeutic effect and can induce side effects such as muscle contractions, ocular deviation, depression and migraine^6^.

Because of its non-invasive nature and unparalleled soft-tissue contrast, magnetic resonance imaging (MRI) with high-field systems (3 T or higher) has great potential for DBS target verification and electrode localization, as well as evaluation of co-morbidities. However, high-field MRI is largely inaccessible to DBS patients at present due to safety concerns^7^, and post-operative MRI at 1.5 T is available only under strict safety guidelines that limit image quality^8^.

The primary safety concern in MRI is the “antenna effect”, whereby the long conductive wires of DBS leads couple with the radiofrequency (RF) electric field transmitted to the patient by the MRI system. Wire currents are produced, terminating in the surrounding tissue to produce a high local specific absorption rate (SAR)^9^. Numerous experiments have been conducted to determine the extent of heating due to coupling between the RF field and DBS implants in both phantom^10,11^ and simulation studies^12–15^. Temperature increases ranging from 1°^16^–46°^17^ have been measured depending on a variety of factors such as magnetic field strength, pulse sequence, DBS lead geometry and the type of RF coil used.

Recent focus on improving the safety for DBS patients during MRI with the goal of reducing coupling between the implants and RF field has taken two approaches: modifying DBS lead design and materials^18–20^, and modifying RF excitation^21,22^. Various techniques have been proposed to tailor the spatial distribution of the RF electric field (E-field) tangential to the implant, thereby reducing coupling and localized heating. Recent techniques include a rotating birdcage coil design^23^ and parallel RF transmission (pTx)^24–26^. Using pTx, the input signal to multiple, independent RF transmitters can be varied to tailor the E-field to reduce coupling with the implant, resulting in reduced heating^27–29^.

Previous pTx studies have included validation experiments that incorporated some elements of real-world DBS scenarios, such as geometrical features of DBS leads (electrode contacts, core and insulation), tissue heterogeneity and unilateral versus bilateral implants^30^. However, the DBS lead trajectory was drastically simplified and each technique was validated on a fixed DBS lead geometry. In reality, DBS leads have complicated trajectories with out-of-plane segments and overlapping loops^31^. The targets of stimulation and trajectory of the implant through the brain are patient- and disease-dependent, resulting in variable implant configurations. Because the trajectory of the implant is an important factor in the extent of heating in the tissue surrounding the implant^17,32,33^, it is essential to investigate the performance of heat-reduction methods in realistic patient-specific scenarios.

The present work addresses this need through further study of the method developed by McElcheran *et al*.^29^, which optimizes pTx to suppress localized heating effects. In the original implementation of the method (which was applied to a unilateral implant) and a subsequent report investigating bilateral implants^30^, optimized pTx was studied for the case of un-insulated, perfectly conducting wires. The present work investigates the more realistic case of insulated implants with exposed conductive tips - a scenario that intensifies localized heating and makes suppression of heating effects more challenging. In addition, the experimental validation of simulations performed in the previous two studies was limited by use of a very basic 4-channel pTx setup that provided rudimentary control of the transmitted phase for each channel, but not the analogous control of amplitude. Here, experimental validation is provided with a more sophisticated pTx setup that includes both amplitude and phase control, and that enables high quality MRI data. The validation is conducted for a challenging case in which optimized pTx must suppress localized heating effects that, if left unchecked, produce large temperature elevations. Lastly, the present work provides the first direct indication that the pTx optimization method is capable of strongly suppressing the localized heating effects produced by the variable implant trajectories that occur in patients. This is achieved through numerical simulations involving the real lead trajectories of nine DBS patients using electromagnetic simulations for MRI at 3 T, with the lead trajectories obtained by image segmentation of intra-operative computed tomography (CT) data of each patient.

## Results

### Local 1g SAR and Localized Temperature Increase

**Figure 1:**
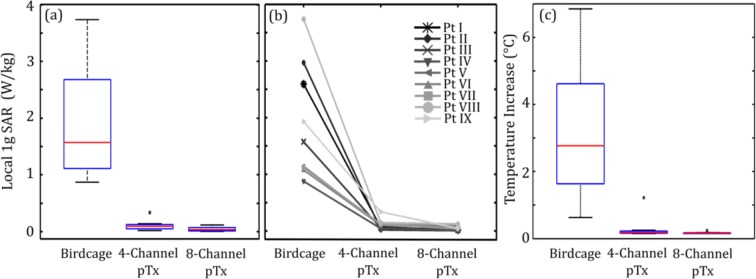
Estimated local 1 g SAR in the ROM surrounding the tip of the DBS electrode for lead trajectories obtained from nine different patients. (**a**) Box and whisker plot showing median, interquartile range (IQR) and data range. Data lying outside 1.5•IQR are shown as outliers. (**b**) Line plot of the individual patient-trajectory data. (**c**) Corresponding peak temperature increase at the tip of the DBS electrode. Results are shown for the birdcage coil operated in quadrature transmission mode and for optimized 4-channel and 8-channel pTx.

Figure 1a shows a box-and-whisker plot of 1 g SAR in the ROM surrounding the DBS electrode tip for the patient-trajectory models. This plot indicates the median value and interquartile range (IQR) of 1 g SAR for the birdcage coil under quadrature transmission, as well as for 4-channel and 8-channel pTx optimized to suppress heating while maintaining B_1_^+^-field homogeneity. Outliers were defined as points lying outside 1.5∙IQR. The median 1 g SAR for birdcage excitation was 1.6 W/kg with an IQR of 1.6 W/kg. The analogous values for optimized 4-channel pTx were 0.10 W/kg and 0.08 W/kg, and for optimized 8-channel pTx were 0.04 W/kg and 0.06 W/kg, respectively. Thus, 4-channel pTx reduced the median 1 g SAR by 94%, whereas 8-channel pTx achieved a reduction of 97%. There was also a statistically significant decrease in the median 1 g SAR for both 4-channel and 8-channel configurations compared with birdcage excitation (Wilcoxon signed rank test, *P* < 0.05). Figure 1b shows a line plot of 1 g SAR for each individual patient for the three coil configurations. Each patient showed a substantial reduction in 1 g SAR when comparing excitation with the birdcage coil to excitation with either optimized pTx coil configuration.

In addition to power deposition, peak temperature elevation at the tip of the lead was calculated for a 180° pulse, analogous to a fast spin echo sequence (Fig. 1c). For birdcage excitation, the median temperature increase was 2.8 °C with an IQR of 2.6 °C. Optimized 4-channel pTx and 8-channel pTx produced significantly lower temperature increases of 0.16 °C with an IQR of 0.05 °C and 0.16 °C with an IQR of 0.02 °C, respectively (Wilcoxon signed rank test, P < 0.05). The birdcage coil produced temperature increases above 1 °C (the limit provided by the IEC^34^) in all but one patient. However, in all 8-channel pTx excitations and all but one 4-channel pTx excitation, the temperature increase was below 1 °C at the tip and was not elevated above background temperature increases throughout the head model.

### B_1_^+^ Inhomogeneity

**Figure 2:**
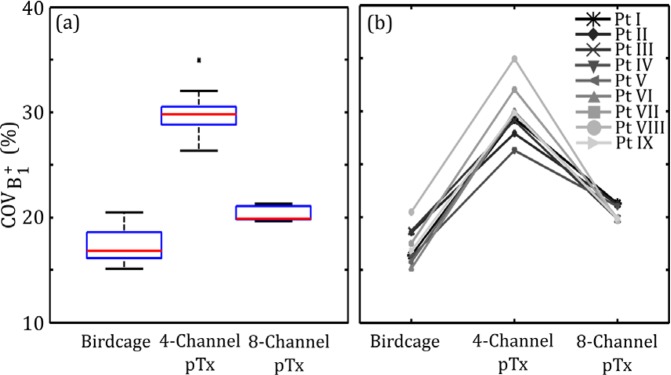
B_1_^+^ inhomogeneity in the VOI for lead trajectories obtained from nine different patients. Results are shown for the birdcage coil operated in quadrature transmission mode and for optimized 4-channel and 8-channel pTx. (**a**) Box and whisker plot showing median, IQR and data range. Data lying outside 1.5•IQR are shown as outliers. (**b**) Line plot of the individual patient-trajectory data.

The B_1_^+^ inhomogeneity results for each coil configuration are shown similarly in Fig. 2a, as quantified by $CO{V}_{{B}_{1}^{+}}$. Birdcage coil excitation produced the most homogeneous field with a median $CO{V}_{{B}_{1}^{+}}$ of 17% and an IQR of 2.5%. The optimized 4-channel pTx produced the worst B_1_^+^ inhomogeneity, with approximately two-fold increase in the median $CO{V}_{{B}_{1}^{+}}$ value of 30% and an IQR of 1.7%. Optimized 8-channel pTx produced an improvement over the 4-channel configuration; however the $CO{V}_{{B}_{1}^{+}}$ values increased (median of 20%, IQR of 1.2%) in comparison with those obtained with the birdcage coil. The increase in median $CO{V}_{{B}_{1}^{+}}$ was statistically significant for both pTx configurations (*P* < 0.05). A line plot of individual paths $CO{V}_{{B}_{1}^{+}}$ values is shown in Fig. 2b. In all models there was a decrease in homogeneity when excitation was completed with 4-channel pTx compared with birdcage excitation. However, in one case there was an increase in homogeneity in 8-channel pTx as compared with birdcage excitation.

### Whole-head Averaged SAR

**Figure 3:**
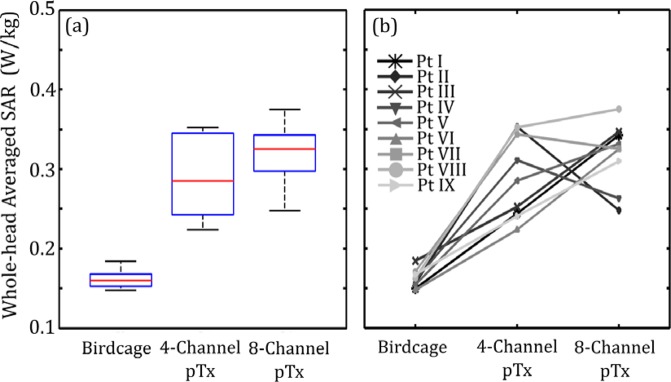
Estimated whole-head averaged SAR for lead trajectories obtained from nine different patients. Results are shown for the birdcage coil operated in quadrature transmission mode and for optimized 4-channel and 8-channel pTx. (**a**) Box and whisker plot showing median, interquartile range and data range. (**b**) Line plot of the individual patient-trajectory data.

The whole-head averaged SAR results are shown in Fig. 3. Although excitation with the birdcage produces high 1 g SAR in the ROM (Fig. 1), background SAR levels throughout the rest of the head were low, leading to a low median SAR_WH_ of 0.16 W/kg and an IQR of 0.02 W/kg. Optimized pTx approximately doubles the whole-head averaged SAR (median SAR_WH_ of 0.29 W/kg and an IQR of 0.10 W/kg for optimized 4-channel pTx; analogous values of 0.33 W/kg and 0.05 W/kg for optimized 8-channel pTx). In both cases of optimized pTx, the increase in median SAR_WH_ was statistically significant (*P* < 0.05), although the difference between 4-channel and 8-channel pTx results was not significant. On further inspection of the simulation results, the increase in SAR_WH_ for optimized pTx comes from slightly more power deposition in background regions. Across all patient models, no new areas of high peak power deposition were generated in regions far from the lead tip by the optimization process.

### Selected Case Results

**Figure 4:**
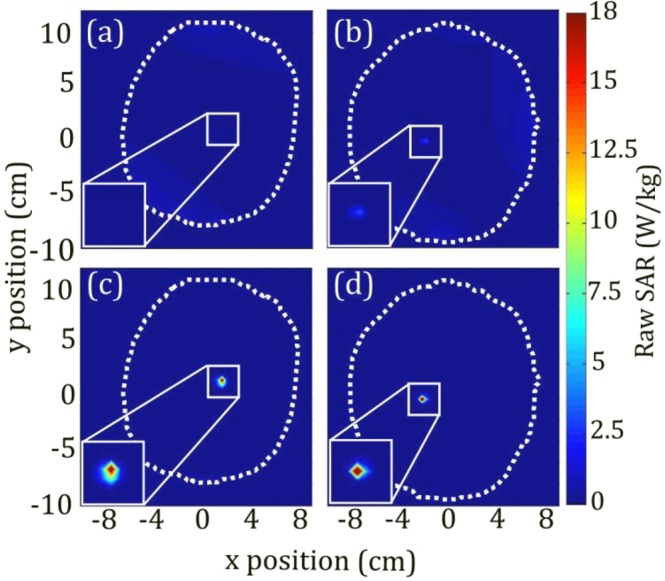
Contour plot of raw SAR (resolution: 0.39 cm × 0.45 cm × 0.35 cm) for (**a**) best solution for local 1 g SAR and (**b**) worst solution for local 1 g SAR with corresponding SAR plots for birdcage excitation (**c**,**d**). White dotted line indicates outline of patient head. Position of plane in z-direction corresponds with maximum SAR location and may differ between plots. Implant region shown at 2x magnification in bottom left corner.

For further demonstration, individual patient cases are shown in Fig. 4, which includes cases with lowest and highest local 1 g SAR in the ROM across both pTx coil configurations (0.008 W/kg in patient II excited with 8-channel pTx and 0.34 W/kg in patient IX excited with 4-channel pTx). The $CO{V}_{{B}_{1}^{+}}$ values for these cases were 21% and 30%, respectively. A contour plot of raw un-averaged SAR with resolution of 0.39 cm × 0.45 cm × 0.35 cm is shown for each case in the axial plane through the tip of the lead, located at *x* = 2 *cm*, *y* = 2 *cm*, where 1 g SAR is of particular concern^35^. In Fig. 4a, a zone of minimal SAR intersects diagonally with the tip of the implant, creating very weak coupling that in practice is indistinguishable from background. The corresponding birdcage excitation of the same patient model is shown in Fig. 4c for comparison, with substantially higher SAR observed surrounding the tip of implant. In Fig. 4b, the “worst” 1 g SAR solution is shown, with a peak raw SAR of 3 W/kg. This is the largest raw SAR value observed within the axial slice. Despite the fact that this value is substantially lower than the maximum raw SAR produced by the birdcage coil for this patient model, namely 24.5 W/kg (Fig. 4d), this solution produces a temperature increase of 1.2 °C.

**Figure 5:**
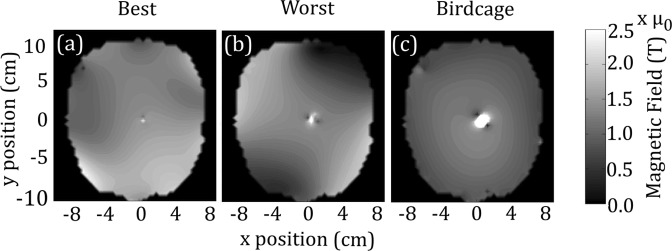
Contour plots of (**a**) best solution for B_1_^+^ inhomogeneity, (**b**) worst solution for B_1_^+^ inhomogeneity, and (**c**) quadrature birdcage excitation.

Similarly, Fig. 5 shows the B_1_^+^-field for the lowest B_1_^+^ inhomogeneity ($CO{V}_{{B}_{1}^{+}}$ = 20%) in Fig. 5a and the highest B_1_^+^ inhomogeneity ($CO{V}_{{B}_{1}^{+}}$ = 35%) in Fig. 5b. The corresponding 1 g SAR values for the lowest and highest B_1_^+^ inhomogeneity solutions were 0.13 W/kg and 0.15 W/kg, respectively. Both cases occur in a single patient model, patient VIII, with the best case occurring when optimization was completed with 8-channel pTx, and the worst case occurring when optimization was completed with 4-channel pTx. In Fig. 5a, the B_1_^+^-field shows excellent homogeneity, with minimal regions of low signal. A small artifact can be seen in the region of the implant tip (at *x* = 0.5 *cm*, *y* = 0.5 *cm*) due to some residual coupling. In Fig. 5b, the inhomogeneity is increased with signal drop off near the edges of the head at approximately *x* = −4 *cm*, *y* = −7 *cm* and *x* = 5 *cm*, *y* = 8 *cm*. An artifact with a higher B_1_^+^-field and larger spatial extent compared with Fig. 5a can be seen at the wire location, due to more coupling with the implant, as seen in the 1 g SAR values (0.15 W/kg vs. 0.13 W/kg). However, the artifact in the worst case scenario (Fig. 5b) is reduced when compared to that obtained with the birdcage excitation (Fig. 5c).

### Validation by Experiment

**Figure 6:**
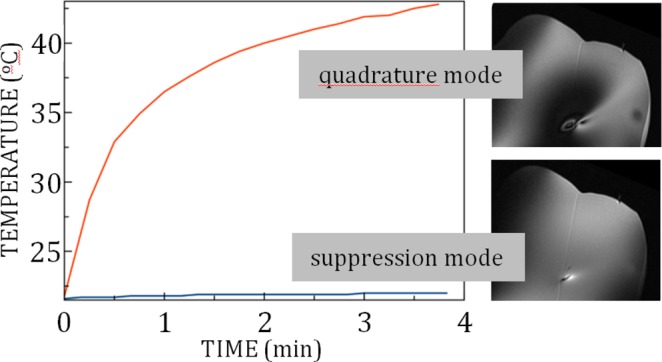
Plots of temperature versus time for pTx TSE MRI in quadrature mode and in suppression mode, recorded at the exposed tip of a wire implanted in a uniform head phantom with a trajectory approximating a high-risk DBS scenario. Images are also shown at the slice location intersecting the wire tip, to evaluate signal uniformity and RF coupling artifacts. Shading at the left side of each image is due to extraneous application of an RF saturation band pulse, not RF coupling. The thin diagonal line shown in both images arose from filling the phantom in two pouring sessions.

Figure 6 shows plots of the 4-channel pTx MRI-induced temperature change at the exposed tip of an insulated wire implanted in a uniform head phantom with a trajectory approximating a high-risk DBS scenario. Image data are also shown at a slice location intersecting the wire tip. Substantial RF coupling and localized heating were generated during TSE imaging in pTx quadrature mode, with the wire tip reaching a temperature of approximately 43 °C at the end of the imaging acquisition. Furthermore, the wire tip did not reach thermal equilibrium during this period. Poor image uniformity was also observed, with bright and dark banding artifacts near the wire tip and a large signal void in the surrounding area. For imaging in pTx suppression mode, the observable effects were much smaller: the temperature elevation was approximately 1 °C and the MRI signal intensity was much more uniform within the slice, with some residual coupling effects observed in close proximity to the wire tip.

### Generic vs. Patient-Specific Solutions

**Figure 7:**
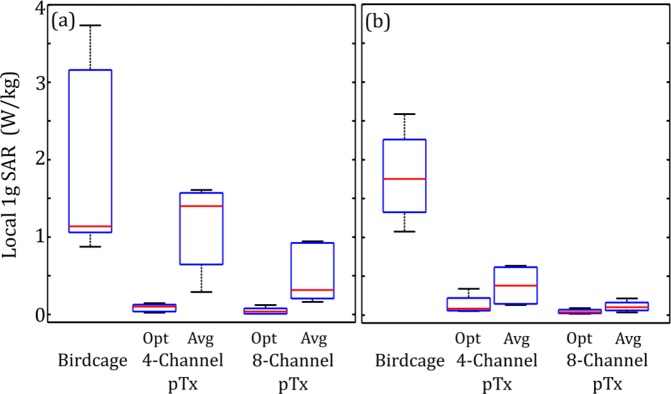
Estimated local 1 g SAR in the ROM surrounding the tip of the DBS electrode for lead trajectories obtained from nine different patients. Results are shown for the birdcage coil operated in quadrature transmission mode and for 4-channel and 8-channel pTx excited with optimized, patient-specific inputs (Opt) and amplitude and phase shifts averaged over patients with the same lateralization (Avg). The box and whisker plots show median, interquartile range and data range for patients with (**a**) left-lateralized implants and (**b**) right-lateralized implants.

Figure 7 shows separate box-and-whisker plots of simulated 1 g SAR for patients with left-lateralized implants and patients with right-lateralized implants, when excitation is performed with generic pTx RF inputs obtained by averaging the patient-specific input amplitude and phase over each group. The 1 g SAR values obtained using patient-specific optimized inputs (as seen in Fig. 1) are included for comparison. The median 1 g SAR was 1.4 W/kg with an IQR of 0.92 W/kg and 0.38 W/kg with an IQR of 0.47 W/kg for 4-channel pTx with averaged inputs in left-lateralized and right-lateralized patients, respectively. Analogous values for 8-channel pTx with averaged inputs were 0.31 W/kg with an IQR of 0.72 W/kg and 0.10 W/kg with an IQR of 0.10 W/kg for left-lateralized and right-lateralized patient groups, respectively. In the left-lateralized group, the averaged inputs produce a statistically significant increase in 1 g SAR for both pTx configurations compared with optimized pTx inputs (Wilcoxon signed rank test, P < 0.05). In addition to median and IQR metrics, the worst case is important to evaluate for safety considerations. In the case of average inputs, the worst cases for the left-lateralized group with 4-channel and 8-channel pTx experience a 1 g SAR that is 137% and 108% of that applied by the birdcage coil, respectively. B_1_^+^ homogeneity remains statistically equivalent to the optimized, patient-specific case with median $CO{V}_{{B}_{1}^{+}}$ of 28% (IQR of 1.5%) and 22% (IQR of 3.3%) for 4-channel and 8-channel pTx excitation, respectively.

### Preliminary Assessment of Position Effects

**Figure 8:**
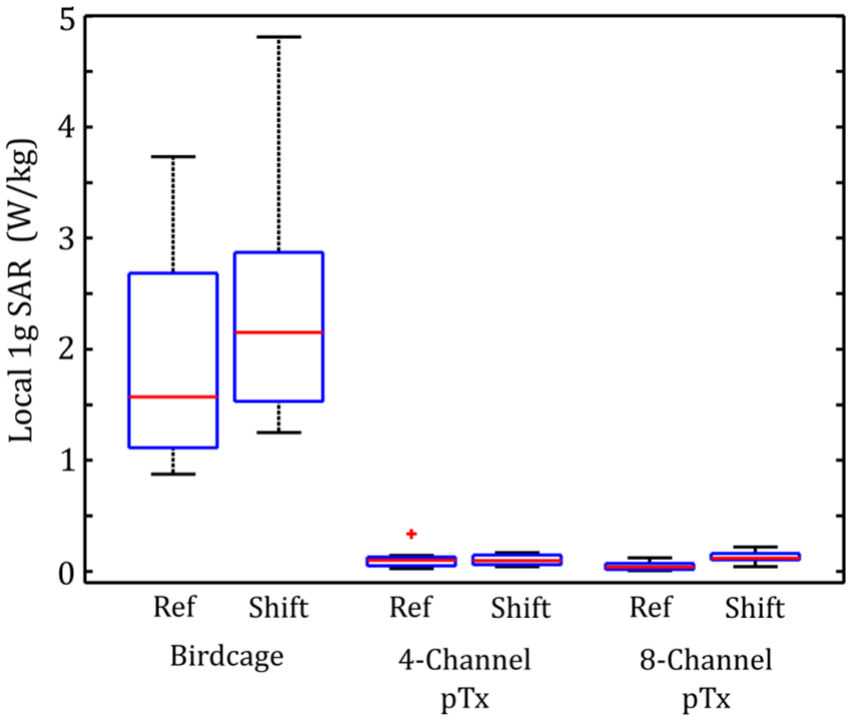
Box and whisker plots showing median, interquartile range (IQR) and data range of estimated local 1 g SAR in the ROM surrounding the tip of the DBS electrode for lead trajectories obtained from nine different patients. Results are shown for original patient-trajectory model orientations and for patient-trajectory models after random rigid-body shift, for the birdcage coil operated in quadrature transmission mode and for optimized 4-channel and 8-channel pTx. Data lying outside 1.5•IQR are shown as outliers.

A box and whisker plot of 1 g SAR for each patient-trajectory model following a random rigid-body displacement, or “shift” in position, is shown in Fig. 8. Original values (as seen in Fig. 1) are included for comparison. For the birdcage coil excitation, median 1 g SAR is increased by shift in comparison to that of the original orientation, to a value of 2.15 W/kg with an IQR of 1.34 W/kg. For the 4-channel pTx configuration, the 1 g SAR after shift (median value of 0.098 W/kg and IQR of 0.088 W/kg) does not show a statistically significant increase compared to that of the original orientation. However, a statistically significant increase in 1 g SAR is observed in the 8-channel excitation between the result after shift (median value of 0.12 W/kg and IQR of 0.056 W/kg) and that of the original orientation. In addition, the shifts had little impact on B_1_^+^ homogeneity across all three coil configurations. For the birdcage coil excitation, the median $CO{V}_{{B}_{1}^{+}}$ is 16% with an IQR of 2.8% for shifted patient-trajectory models. Analogous values for 4-channel pTx and 8-channel pTx are 30% with an IQR of 2.4% and 21% with an IQR of 1.7%, respectively.

## Discussion

This study provides important evidence that pTx RF shimming can be optimized to minimize heating at DBS lead tips when different realistic lead trajectories are taken into account. For the lead trajectories of nine DBS patients, optimized RF shimming simulations resulted in a significant decrease in median local 1 g SAR of 94% for 4-element pTx and 97% for 8-element pTx, as well as a decrease in 1 g SAR for every trajectory. In addition, although B_1_^+^ homogeneity decreased compared with birdcage excitation, both pTx configurations produced reasonable homogeneity over a large field of view throughout the head. Furthermore, the simulation results were reinforced by an example validation experiment with 4-channel pTx MRI with a power-intensive TSE sequence, which showed the effectiveness of suppression mode to suppress RF coupling and localized heating effects produced at the exposed tip of an insulated wire in a uniform phantom, with the wire implanted to approximate the lead trajectory of an actual DBS patient.

The adopted approach to optimizing RF shimming inputs does not provide analytic, closed-form solutions. Instead, a cost function is optimized that simultaneously minimizes power deposition within a ROM and B_1_^+^-field inhomogeneity within a VOI. This approach naturally leads to reporting of power deposition over the ROM, in terms of 1 g SAR values, but this raises a potential concern that the volume averaging inherent to the 1 g SAR calculation could under-represent extremely localized power deposition and related temperature elevations immediately adjacent to the tip of the DBS lead. After investigating raw SAR at relatively high spatial resolution, and investigating the potential temperature elevations produced by select RF delivery parameters using the Pennes bioheat transfer equation, this concern appears to be unfounded. Raw SAR values and peak temperature elevations observed at the lead tip were modest for almost all patients with optimized 4-channel and 8-channel pTx. Only one case involving 4-channel optimized pTx resulted in a temperature elevation exceeding 1 °C. Thus, the reporting of results in terms of 1 g SAR values is not obscuring potentially unsafe heating conditions in the present context when pTx is optimized on a patient-specific basis. For brevity, 1 g SAR values have been supplemented by the available raw SAR values and temperature calculations where necessary.

The 8-element pTx configuration performed better than the 4-channel pTx configuration. The 1 g SAR value was significantly improved from 0.1 W/kg for the 4-channel case to 0.04 W/kg for the 8-channel case, and in analogous fashion the group median $CO{V}_{{B}_{1}^{+}}$ improved from 30% to 20%. There was one case for 4-channel pTx where simulation results indicated temperature elevations at the lead tip that exceeded 1 °C. Overall, this suggests that 1) the additional degrees of freedom provided at higher channel count are an important resource when actual DBS lead trajectories are considered; and 2) improved methodology will be required to expand pTx capabilities at low channel count for suppressing localized heating effects safely across DBS patient populations. It will be important to consider the latter issue, as 2-channel pTx is available on newer 3 T MRI systems. The present work did not quantitatively evaluate 2-channel pTx because of previous numerical simulation results that show insufficient degrees of freedom are available in that configuration to suppress localized heating without substantially attenuating B_1_^+^ (and thus badly degrading image quality) near the tip of the implant^29^. Nevertheless, there is still the alternative to take advantage of the current commercially available, standard-of-care imaging systems: for example, to perform pre-operative MRI; obtain CT images of the implanted DBS device; conduct simulations and predict possible unsafe conditions; and undertake countermeasures as appropriate, such as subsequent MRI of the DBS patient with a very low-power protocol.

One limitation of using the pTx approach to minimize local SAR surrounding the implant tip is an increase in whole-head averaged SAR. The simulation results showed that pTx optimizations produced approximately twofold increase in SAR_WH_, and effective suppression of localized heating (in all but one case) for SAR_WH_ values that are slightly greater than the 0.1 W/kg limit set for some MRI-conditional DBS devices^36^. This suggests that there may be additional “headroom” for optimized pTx to be conducted for higher values of SAR_WH_, closer to the (much less restrictive) limits for head imaging of patients without brain implants. Such a scenario has the potential to provide considerably more flexibility in MRI protocols for DBS patients, but ultimately will require careful verification imaging *in vivo*.

A simple method for generating generic input weights was investigated to evaluate the suitability of “one size fits all” solutions. Patient-trajectory models were grouped by lateralization of implant to limit differences in lead tip location and input weights for each group were averaged and applied to all patient-trajectory models in the group. This approach reduced the median local 1 g SAR by 61% and 86% compared to birdcage excitation for 4-channel and 8-channel pTx for both groups. In the worst case, however, local 1 g SAR was raised by 137% for 4-channel excitation and 108% for 8-channel excitation when compared to patient-specific input weights. In addition, the range of 1 g SAR values increased compared with the patient-specific optimization, yielding 1 g SAR values that were larger than those created with birdcage excitation. Given that an actual implementation will necessitate safe MRI for every DBS patient, the present work suggests that patient-specific inputs are necessary to utilize this pTx methodology.

Preliminary simulations were also conducted to investigate whether optimized pTx solutions involving a specific patient and lead trajectory are robust to small changes in head position and orientation that could occur between calculation of an optimized solution and actual imaging. Randomized rigid-body transformations (shifts in position and angular orientation) were applied to the patient-trajectories and optimized input weights were applied as derived from the original patient-trajectories. The resulting variations in 1 g SAR were quite small, with a statistically significant increase in 1 g SAR compared with the ideal solutions for 8-element pTx and no statistically significant change in 1 g SAR compared with ideal solutions for 4-element pTx. The levels of B_1_^+^ inhomogeneity were not significantly affected in all three coil configurations. These results suggest that a small deviation in patient position may be acceptable, contingent on the spatial extent that the E-field can be minimized near the DBS lead tip. However, further systemic investigation is necessary for a complete understanding of the effects of model/patient deviations.

In addition, there are numerous other parameters of interest that require further investigation as part of assessing the robustness of this pTx methodology, including the uncertainty in RF shim parameters (prescribed versus delivered), sensitivity to DBS target location, and magnetic field strength. Such investigations are beyond the scope of the present work, although it is reasonable at present to advocate for development of on-line safety checks to ensure that patient/model errors do not lead to unsafe MRI conditions. Promising approaches include monitoring the MRI signal artifacts at the lead tip as a proxy for RF coupling levels and the associated E-fields^37^, and development of on-line B_1_-mapping techniques using optimized inputs to ensure low coupling with the implant^38^.

Although the results of the present study are promising, considerable work still remains to develop a robust clinical implementation. For example, intra-operative CT scans are obtained prior to the implantation of the power generator (IPG), which is connected to the loose wires and implanted in the chest or abdomen. Implanting the IPG has been known to change the lead trajectory and therefore future investigations into the extent of the change in lead trajectory due to IPG implantation are necessary. Possible solutions such as post-implantation CT or collaborative efforts with surgeons to obtain final lead positioning at the intra-operative stage may be investigated. As well, patient models do not currently contain the shoulders, as the shoulders are not included in the clinical post-operative imaging protocol. In future collaborations, physicians may be informed of the desire to extend the CT field of view and thus allow for head and shoulder models of patients. This would enable exploration of the MRI-related RF coupling effects produced with DBS devices from the lead tip to the neurostimulator, and the ability to suppress localized heating using pTx in this scenario. Deviations from the results of the present work are expected to be minor, however. Finally, deviation between simulation and real-life scenarios must be minimized and monitored to ensure safe scanning. Scan-time modifications of pTx input and reliable scan-time “checks” should be implemented before clinical implementation can be achieved.

This investigation shows the robustness of the pTx method to reduce heating in DBS leads over a variety of lead trajectories. Statistically significant reductions have been made in nine patient models with both 4-element and 8-element pTx configurations as compared with a standard T/R birdcage coil, while reasonable transmit field homogeneity was maintained.

### Disclaimer

The mention of commercial products, their sources, or their use in connection with material reported herein is not to be construed as either an actual or implied endorsement of such products by the Department of Health and Human Services.

## Materials and Methods

### Optimized RF Shimming

RF shimming is a subset of pTx that utilizes a common input signal to each of the parallel RF channels weighted with a static amplitude *A*_*n*_ and phase shift *φ*_*n*_^39^. The RF fields of interest for MRI are the transmit B-field (B_1_^+^), the circularly polarized phasor of the magnetic field which is resonant with the nuclear spins; and the E-field, which is related to SAR by:

1 $$SA{R}_{volume}=\frac{1}{V}{\int }_{volume}\frac{\sigma ({\boldsymbol{r}})\cdot {|{\boldsymbol{E}}({\boldsymbol{r}})|}^{2}dv}{2\cdot \rho ({\boldsymbol{r}})}$$

where *σ*(***r***) is the conductivity of the medium at position ***r***, *ρ*(***r***) is the density of the medium at ***r***, *V* is the total volume of interest and |***E***| is the magnitude of the E-field phasor at ***r***^40^. Local 1 g SAR is defined according to eq. (1) where the volume is defined as a cubic region containing 1 g of tissue.

The **E**- and B_1_^+^-fields created during RF shimming are related to the field profile of the individual coil element and the amplitude and phase shift applied to the input signal. In phasor notation, the total fields ***E***_***tot***_(***r***) and ${B}_{1,tot}^{+}({\boldsymbol{r}})$ are given by:

2 $${{\boldsymbol{E}}}_{{\boldsymbol{tot}}}({\boldsymbol{r}})=\sum _{N}{A}_{n}{e}^{i{\phi }_{n}}{{\boldsymbol{e}}}_{{\boldsymbol{n}}}({\boldsymbol{r}})$$

3 $${B}_{1,tot}^{+}({\boldsymbol{r}})=\sum _{N}{A}_{n}{e}^{i{\phi }_{n}}{b}_{1,n}^{+}({\boldsymbol{r}})$$

where ${b}_{1,n}^{+}$ is the b_1_-map for coil *n*, and ***e***_***n***_ is the **e**-map for coil *n*, and *N* is the total number of coils. A b_1_-map and **e**-map are the B_1_^+^-field and **E**-field, respectively, throughout space generated by a particular coil when excited with a constant input voltage (for simulation purposes, an input voltage of 1 V is used) with remaining coil inputs set to zero. If the b_1_-maps and **e**-maps are known for all coils, then the input amplitude (*A*_*n*_) and phase (*φ*_*n*_) can be varied to achieve a desired spatial distribution of ***E***_***tot***_ and ${B}_{1,tot}^{+}$. The **E**-field and B_1_^+^-field distributions of interest endeavour to minimize heating in the implant by suppressing the **E**-field in pertinent regions near the implant, while maintaining spatially homogeneous B_1_^+^-field for good image quality.

A simplex optimization algorithm^41^ is used to determine the optimal amplitude and phase for each channel, via custom scripts in Matlab (The Mathworks Inc., Natick, MA). Two separate sets of regions are defined for the optimization: a volume of interest (VOI) and regions of minimization (ROMs). The VOI is defined as the region over which an image is required. In this region, B_1_^+^ inhomogeneity is minimized to provide reasonable flip angle uniformity during excitation. The ROMs are a set of regions located at areas that produce high E-field in the un-optimized scenario when the RF field couples strongly with the device (e.g., surrounding the tip of the implant). The cost function is defined as:

4 $$mi{n}_{{{A}}_{{n}},{{\phi }}_{{n}}}(\frac{{{s}}_{{VOI}}(|{{B}}_{1,{tot}}^{+}|)}{{{\mu }}_{{VOI}}(|{{B}}_{1,{tot}}^{+}|)}+{\lambda }\cdot {{\mu }}_{{ROM}}(\frac{{|{{E}}_{{tot}}|}^{2}}{{|{{E}}_{{quad}}|}^{2}}))$$

where ***s***_***VOI***_(·) is the standard deviation over the VOI; *μ*_*VOI*_(·) and *μ*_*ROM*_(·) are the mean over the VOI and the ROM, respectively; ***λ*** is a weighting factor used to control the relative importance of safety (through the E-field term) and image quality (through the B_1_^+^-field term); and ***E***_***quad***_ is the E-field in pTx quadrature mode (*A*_*n*_ = 1, *φ*_*n*_ = 2*πn*/*N*).

Prior to optimization, *μ*_*ROM*_(|*E*_*quad*_|^2^) is evaluated to determine if the quadrature mode produces minimal local heating at the implant tip. If *μ*_*ROM*_(|*E*_*quad*_|^2^) is less than a predetermined safety limit, chosen as *SAR*_*ROM*_ = 0.4 *W*/*kg*, the quadrature mode is deemed safe and the optimization is not performed. This is done to ensure that the case where *μ*_*ROM*_(|*E*_*quad*_|^2^) approaches zero is not included in the optimization.

The appropriate value of the weighting factor (λ) should be chosen on a subject-by-subject basis, as this parameter provides an additional degree of freedom leading to improved solutions. The weighting factor is chosen to ensure 1) that local 1 g SAR in ROMs does not exceed a pre-determined safety limit; and, once the safety limit has been enforced, to ensure 2) that optimal B_1_^+^ inhomogeneity is maintained. This is achieved by first performing a set of optimizations with weighting factors extending over a range that covers solutions solely dependent on B_1_^+^ homogeneity (at *λ* = 0) until the E-field reduction and B_1_^+^ homogeneity no longer vary with increasing λ (at approximately *λ* = 6). The two terms of the cost function are then evaluated for each optimization to choose the appropriate λ value. First, a local SAR limit is set and all solutions exceeding this threshold are eliminated. The present work used a limit of *SAR*_*ROM*_ = 0.4 *W*/*kg* for demonstrative purposes which represented a 75% decrease in 1 g SAR compared with the median birdcage coil excitation. Second, a limit on the B_1_^+^ inhomogeneity is set at 10%. If there are solutions that fit this criterion, then the solution with the minimum 1 g SAR is chosen. If no solutions are within this limit, the maximum B_1_^+^ inhomogeneity is increased by 5% and the solution space is searched again for acceptability. This is repeated until at least one solution meets the maximum B_1_^+^ inhomogeneity.

### Simulation Model

Electromagnetic (EM) fields were calculated using FEKO simulation software (Altair, MI, USA) which formulates EM fields through a hybrid of Method of Moments (MoM) and Finite Element Method (FEM)^42,43^. The EM fields were calculated on a 3-dimensional grid in the VOI and ROMs, providing B_1_^+^-field and E-field values at each point on the grid, creating the b_1_-map and e-map for each coil element. A Hampel filter was applied to the simulation data to remove non-physical spikes that were present at dielectric interfaces due to mesh segmentation. The spatial resolution for all simulations was 0.39 cm × 0.45 cm × 0.35 cm.

**Figure 9:**
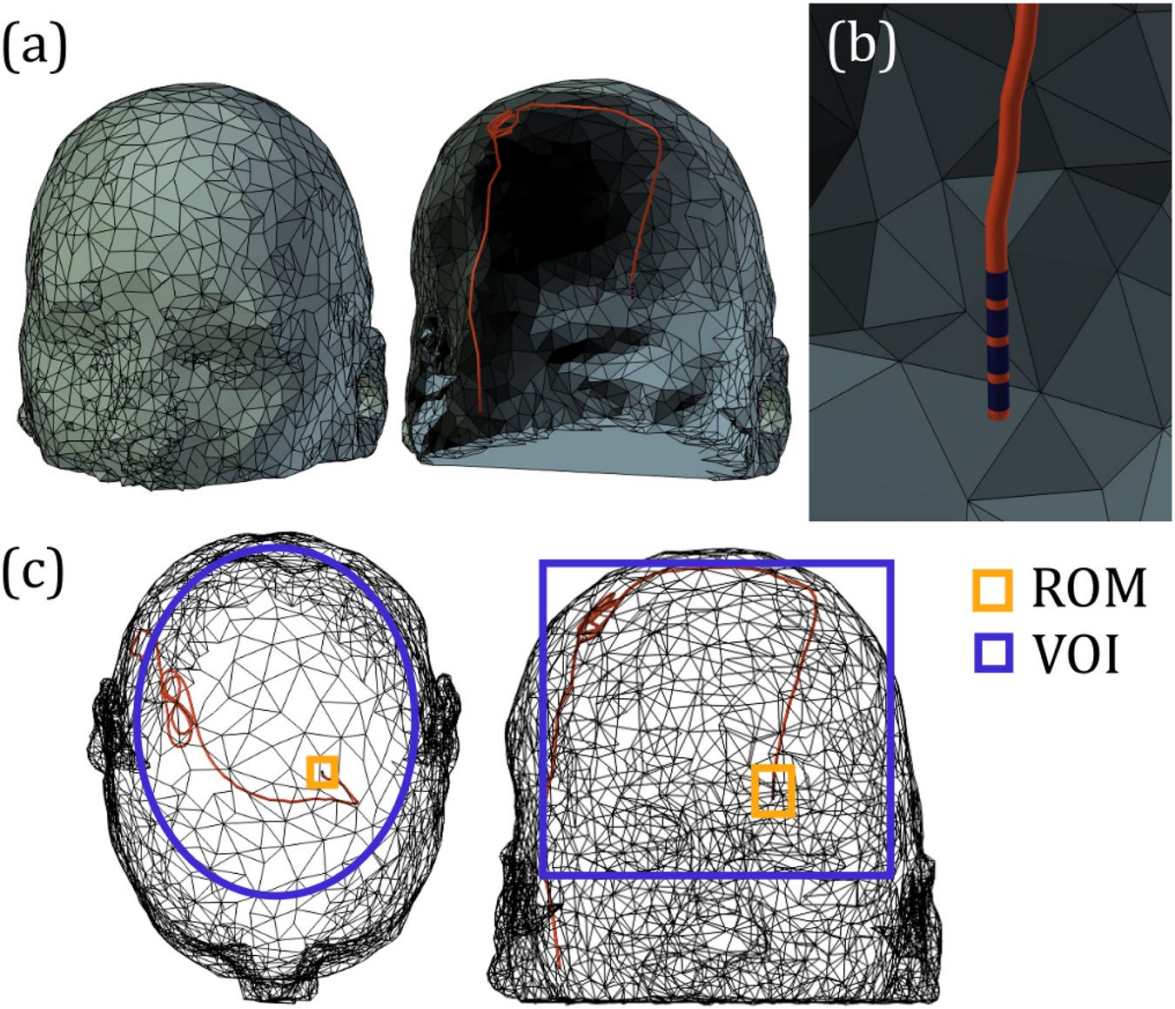
(**a**) Example patient head mesh model with implanted DBS lead trajectory. (**b**) Zoomed-in view of the DBS target location showing wire insulation in red and four exposed electrodes in dark blue. (**c**) Transparent view of the model showing the region of minimization (ROM) for minimizing local power deposition (yellow) and the volume of interest (VOI) for minimizing B_1_^+^ inhomogeneity (blue.).

The VOI was chosen as an elliptical column centered at *x* = −0.5 *cm*, *y* = 1 *cm* and *z* = 3 *cm* where the origin was located at the center of the coil assembly. The radii of the VOI were *r*_*x*_ = 6 *cm* along the x-axis and *r*_*y*_ = 7.5 *cm* along the y-axis. The length of the VOI along the z-axis was *L* = 4.5 *cm*. This region corresponds to the approximate location of the brain when a subject is positioned in the center of the coil. The ROM was chosen as the region of 1 g of tissue surrounding the un-insulated portion of the implant, at the tip located within the brain. This location was defined for each individual subject. An example of the VOI and ROM super-imposed on a representative patient model is shown in Fig. 9.

Potential temperature increases due to RF power deposition were calculated in FEKO using a finite-difference solution of Pennes bioheat transfer equation^44^:

5 $${{\rho }}_{{t}}{{C}}_{{t}}\frac{\partial {T}({r},{t})}{\partial {t}}={{k}}_{{t}}{{\nabla }}^{2}{T}({r},{t})+{{V}}_{{b}}{{\rho }}_{{b}}{{C}}_{{b}}({{T}}_{{b}}-{T}({r},{t}))+{{\rho }}_{{t}}{SAR}({r})+{{Q}}_{{m}}$$

where T is the temperature, *t* is the time, *ρ*_*t*_ is the tissue density, *C*_*t*_ is the specific heat of the tissue, *k*_*t*_ is the thermal conductivity of the tissue, *V*_*b*_ is the perfusion rate per unit volume of tissue, *ρ*_*b*_ is the blood density, *C*_*b*_ is the specific heat of blood, *T*_*b*_ is the temperature of blood and *Q*_*m*_ is the metabolic rate. On the right-hand side of the equation, the thermal diffusion is modelled by the first term; perfusion is modelled by the second term, the third term models heat deposition from the RF field and the fourth term models metabolic heat deposition. Initial body temperature was 37 °C with an ambient temperature of 20 °C.

Patient models consisted of a homogeneous head volume with tissue mimicking electromagnetic properties at 128 MHz, permittivity of ${\epsilon }_{{r}}=67$ and conductivity of *σ* = 0.69 *S*/*m*^45^, and a DBS implanted lead. Thermal properties of the head volume were modelled after average values for brain tissue and blood from the literature^46^. Specific heats of *C*_*t*_ = 3650 *W*/*kg* · *K* and *C*_*b*_ = 3617 *W*/*kg* · *K* were used for brain tissue and blood, respectively. The thermal conductivity of tissue was *k*_*t*_ = 0.6 *J*/*m* · *K*, blood perfusion rate was *V*_*b*_ = 0.0085 *kg*_*blood*_/*s*/*kg*_*tissue*_ and blood temperature was constant at *T*_*b*_ = 37 °C. A uniform head volume was used to evaluate the specific contributions to heating from variability in the lead trajectory across patients, complimentary to previous work investigating the effects of tissue heterogeneity^47^.

The implant was modelled as an insulated lead with four exposed cylindrical electrodes at the tip (Fig. 9b). The permittivity and conductivity of the insulation were ${\epsilon }_{{r}}=3.5$ and *σ* = 10^−10^ *S*/*m*^46^, respectively, and the wire was assumed a perfect electrical conductor. The specific heat of the insulation was 1500 *J*/(*kg* · *K*) and the thermal conductivity was 0.026 *J*/(*m* · *K*)^46^. No metabolic heating or perfusion was modelled within the insulation. The patient model was centred within each coil configuration and oriented such that the head was parallel to the z-axis with the patient facing the negative y-axis.

**Figure 10:**
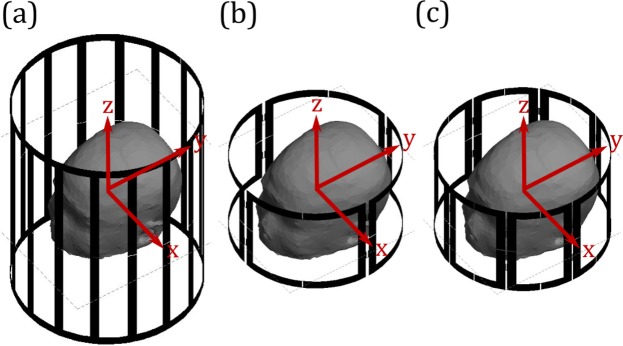
Coil configurations for (**a**) birdcage excitation, (**b**) 4-element pTx excitation and (**c**) 8-element pTx excitation. The Cartesian coordinate system (red) and an example of a patient model are also shown.

Three coil assemblies were investigated: a standard transmit/receive (T/R) birdcage head coil, a 4-element pTx coil and an 8-element pTx coil (Fig. 10). The T/R birdcage coil was modelled after the coil provided on a local research-dedicated 3 T MRI system (MR750, GE Healthcare, Waukesha, WI). This birdcage coil is a low-pass configuration with 16 rungs, a diameter of 28 cm and height of 35 cm. The pTx coils were placed around a cylinder with diameter of 25 cm and a height of 15 cm. The coil elements were located 2***π***/***N*** radians apart with an arc length for each coil of 19.6 cm in the 4-element configuration and 9.8 cm in the 8-element configuration. The pTx coil sizes and configurations were chosen to minimize B_1_^+^ inhomogeneity when operating in quadrature mode, resulting in a different coil diameter compared to the birdcage coil. All coils were tuned and matched to a 50 Ω source while loaded with a head-shaped homogeneous test medium with electromagnetic properties equivalent to the patient models. Coil elements in the pTx configurations were decoupled via capacitive decoupling. Tuning, matching and decoupling capacitor values were determined in simulation through circuit analysis. The residual coupling between coil elements was included when determining the optimal amplitude and phase inputs, according to optimization procedure described above. To facilitate comparison across different coil configurations, input voltages were scaled such that the average B_1_^+^-field over the VOI was $1\,\cdot \,{{\mu }}_{0}$.

### Patient-derived lead models

Realistic DBS lead trajectories and head models were semi-automatically segmented from intra-operative CT images of patients after undergoing surgery for sub-thalamic nucleus DBS implantation at Massachusetts General Hospital (MGH). Secondary use of patient data for modeling and simulation of DBS leads was approved by the Internal Review Board of MGH. Patients provided their free and informed consent to participate by providing their lead trajectory data for the research, which was conducted in accordance with all relevant guidelines and regulations.

**Figure 11:**
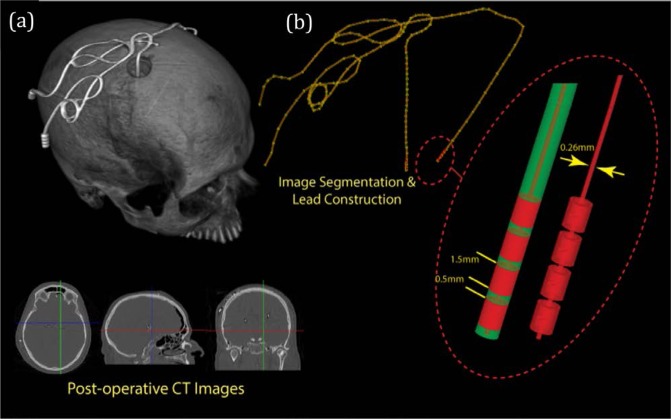
(**a**) Three-dimensional reconstruction of intra-operative CT images from a DBS patient, with representative axial, coronal and sagittal images shown below. (**b**) Segmentation of DBS lead, with zoomed-in region showing in detail how the lead tip was modelled.

**Figure 12:**
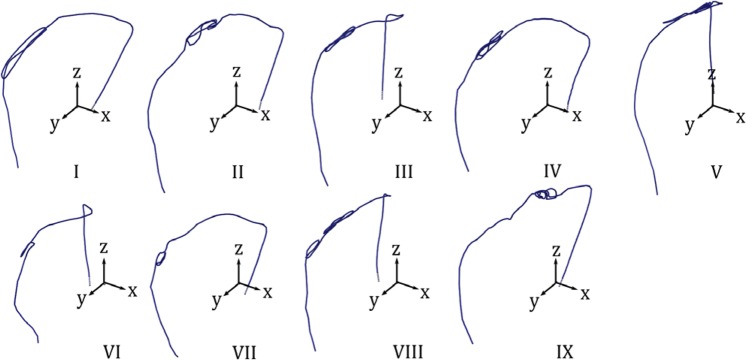
Lead trajectories for nine patient models (patient numbers I–IX).

A thresholding algorithm based on an intensity histogram analysis was applied to extract the silhouette of the head and a preliminary mask of the hyperdense DBS lead from the CT images (Fig. 11). The masks of the leads contained several topological defects due to the presence of self-intersecting sections and overlapping loops, requiring further image processing. First, the centerline of the lead was estimated using a skeletonization algorithm followed by a smooth curve-fitting algorithm (Simpleware Ltd., UK). The intersecting segments of the centerline were then manually adjusted to assure a minimum distance between segments greater than the lead diameter (1.27 mm). The lead trajectories were initially segmented for each patient and then finalized by adding models of the electrode contacts, core, and insulation. Models were composed of four cylindrical contacts (outer diameter = 1.27 mm, wall thickness = 150 µm), connected through a solid straight central core (diameter = 260 µm). This model is approximate as in reality, each electrode is connected to an individual wire. In total, nine patient head models were investigated with varying lead orientation. There were four right-lateralized and five left-lateralized implant-trajectories, with lead lengths ranging from approximately 40–50 cm (Fig. 12).

### Statistical Analysis

Optimized inputs were calculated for each patient-trajectory model in both the 4-element and 8-element pTx configurations. The E-field and B_1_^+^-field generated by the optimized inputs were calculated in Matlab and the resultant SAR (derived from the E-field), temperature increase and B_1_^+^-field were compared across the two pTx implementations (4-element and 8-element) and with the fields generated using a standard birdcage coil. Four metrics were investigated to assess the effects of RF transmission for each coil configuration across the group of patient models. First, the 1 g SAR was quantified in the ROM. Second, the coefficient of variation of the B_1_^+^-field, ${CO}{{V}}_{{{B}}_{1}^{+}}$, was calculated within the VOI to quantify inhomogeneity according to the first term in the cost function:

6 $${CO}{{V}}_{{{B}}_{1}^{+}}=\frac{{{s}}_{{VOI}}(|{{B}}_{1,{tot}}^{+}|)}{{{\mu }}_{{VOI}}(|{{B}}_{1,{tot}}^{+}|)}.$$

Third, the whole-head averaged SAR (SAR_WH_) was calculated. As this quantity was not included in the cost function, it was important to assess whether the optimized pTx solutions were achieved at the expense of deleterious elevations in whole-head averaged SAR. Finally, peak temperature increase at the tip of the lead was calculated. This enabled investigation of the impact of spatial averaging over the ROM during the optimization procedure, evaluation of the appropriateness of using the 1 g SAR metric in the current context, and direct estimation of a quantity more directly linked to biological effects. For each metric, statistically significant group differences across each coil configuration were determined using a Wilcoxon signed rank test with a confidence interval of 95%.

### Example Validation Experiment

To ensure that the simulation results were reliable, and to justify using the simulations to make predictions (see Robustness below), an example validation experiment was conducted using custom 4-channel pTx hardware integrated with a research-dedicated 3 T MRI system (Magnetom Prisma, Siemens, Erlangen, Germany)^47^. A transceiver (identical in geometry to that described for Fig. 9b) was fabricated and connected to a specialized coil adapter (Stark Contrast GmbH, Erlangen, Germany) with 4 preamplifiers and transmit/receive switches, enabling multicoil transmit and receive. The adapter also externalized the high power RF transmit line from the standard Prisma hardware, which was run through a directional coupler to a single channel receive coil and phantom located remotely from the magnet. This “dummy load” pathway allowed the MRI system to pass all software and hardware checks while in PTX mode. The directional coupler also attenuated the transmitted RF in a separate signal pathway back to the equipment room through the penetration panel of the RF shield, providing an appropriate low-amplitude RF input source for a 4-channel phase and gain controller (CGP-128-4C, CPC, Hauppauge, USA) to enable RF shimming. The resulting RF shimmed waveforms each passed through one of four separate RF power amplifiers (BT01000-AlphaSA, Tomco Technologies, Stepney, AUS) to drive the transceiver coil. The amplifiers were RF-gated by passing the appropriate MRI system logic signals through an optical to TTL converter (AFBR-26 × 4Z, Broadcom, San Jose, USA).

An insulated 61 cm copper wire with an exposed tip was placed in a uniform head phantom to approximate the lead trajectory that produced the largest 1 g SAR value in simulation, for excitation with the birdcage coil (patient 8). The trajectory included the looped four-turn figure-eight pattern representative of how excess lead length was bundled at the skull by the neurosurgeon. A simulation study has recently demonstrated that the loop geometry is a very important factor influencing the extent of RF coupling during MRI^48^. The penetrating portion of the lead conformed closely to the specific patient model. A fibre-optic probe (OTg-MPK5, Opsens Inc., Quebec City, Canada) was also attached to the lead tip to measure temperature elevations during subsequent pTx MRI experiments. The head phantom was filled with a 4.5 L oil-in-gelatin mixture to mimic gray matter tissue and its corresponding electromagnetic properties (oil = 3%, NaCl = 4.5 g/L, gelatin = 170 g/L)^49^. For comparison purposes, pTx MRI was performed in two modes: “quadrature mode” to produce strong RF coupling effects, with identical RF pulse amplitude for each channel and a phase shift of 90° between neighbouring channels; and “suppression mode”, to reduce RF coupling to minimal levels, with RF shim settings optimized for the specific lead trajectory. Imaging was performed using a turbo spin-echo sequence with high power deposition (acquisition matrix = 256 × 205, in-plane resolution = 0.5 mm × 0.5 mm, TR/ TE = 516 ms/6.7 ms, slice thickness = 2 mm, acquisition time = 3.8 mins).

### Robustness

From a practical standpoint, some deviation between the results of electromagnetic simulations and actual MRI experiments is inevitable. In the case of heating in DBS leads, this deviation has the potential to create un-safe situations for patients. Extensive study of the safety of any solution under a variety of test conditions is necessary before implementation in clinic to ensure patient safety. Although these investigations are beyond the scope of this work, a preliminary investigation of robustness is presented here for context.

One important question is whether a “one size fits all” approach is possible, in which optimized pTx inputs can be developed for groups of patients that have similar DBS targets. An initial evaluation into whether this has merit, or whether patient-specific solutions are advisable, was achieved by applying average input values to groups of patients and comparing the results with those obtained with optimal inputs generated for each particular patient. Patient-trajectory models were grouped according to the lateralization of the implant (four right-lateralized and five left-lateralized). This was done to minimize the displacement of the implanted lead between patient-trajectory models in each group. For each group, the optimal input amplitudes and phases were averaged for each input channel, respectively. These average amplitudes and phases were then applied to each patient-trajectory model and the 1 g SAR in the ROM and ${CO}{{V}}_{{{B}}_{1}^{+}}$ in the VOI were calculated.

Deviations in head position between simulation and experiment are also a potential source of error which may compromise safety. A preliminary investigation of the effect of rigid-body displacement of the patient-trajectory model was completed by applying a random rotation (maximum range of 10° about each axis) and translation (maximum range of 4 cm) to each patient-trajectory model and applying the input weights obtained for the original orientation. Local 1 g SAR in the ROM and ${CO}{{V}}_{{{B}}_{1}^{+}}$ in the VOI were evaluated for each coil configuration.
